# ﻿Taxonomy and phylogeny of the novel rhytidhysteron-like collections in the Greater Mekong Subregion

**DOI:** 10.3897/mycokeys.86.70668

**Published:** 2022-01-12

**Authors:** Guang-Cong Ren, Dhanushka N. Wanasinghe, Rajesh Jeewon, Jutamart Monkai, Peter E. Mortimer, Kevin D. Hyde, Jian-Chu Xu, Heng Gui

**Affiliations:** 1 Center of Excellence in Fungal Research, Mae Fah Luang University, Chiang Rai 57100, Thailand; 2 School of Science, Mae Fah Luang University, Chiang Rai 57100, Thailand; 3 Guiyang Nursing Vocational College, Guiyang 550081, Guizhou, China; 4 Department of Economic Plants and Biotechnology, Yunnan Key Laboratory for Wild Plant Resources, Kunming Institute of Botany, Chinese Academy of Sciences, Kunming 650201, China; 5 Center for Mountain futures, Kunming Institute of Botany, Chinese Academy of Sciences, Honghe County 654400, Yunnan, China; 6 World Agroforestry Centre, East and Central Asia, Kunming 650201, Yunnan, China; 7 Department of Health Sciences, Faculty of Medicine and Health Sciences, University of Mauritius, Reduit, Mauritius

**Keywords:** Ascomycota, one new taxon, phylogeny, saprobic, taxonomy, Yunnan

## Abstract

During our survey into the diversity of woody litter fungi across the Greater Mekong Subregion, three rhytidhysteron-like taxa were collected from dead woody twigs in China and Thailand. These were further investigated based on morphological observations and multi-gene phylogenetic analyses of a combined DNA data matrix containing SSU, LSU, ITS, and *tef*1-α sequence data. A new species of *Rhytidhysteron*, *R.xiaokongense* sp. nov. is introduced with its asexual morph, and it is characterized by semi-immersed, subglobose to ampulliform conidiomata, dark brown, oblong to ellipsoidal, 1-septate, conidia, which are granular in appearance when mature. In addition to the new species, two new records from Thailand are reported viz. *Rhytidhysterontectonae* on woody litter of *Betula* sp. (Betulaceae) and Fabaceae sp. and *Rhytidhysteronneorufulum* on woody litter of *Tectonagrandis* (Lamiaceae). Morphological descriptions, illustrations, taxonomic notes and phylogenetic analyses are provided for all entries.

## ﻿Introduction

Hysteriaceae was introduced by Chevallier (1826) with *Hysterium* as the type genus, which was characterized by hysterothecial or apothecioidal, carbonaceous ascomata with a pronounced, longitudinal slit running the length of the long axis, 8-spored, clavate to cylindric asci with an ocular chamber as well as obovoid, clavate, ellipsoid or fusoid, hyaline to light- or dark brown, one to multi-septate or muriform, smooth-walled ascospores with or without a sheath (Boehm et al. 2009b; Hongsanan et al. 2020; Hyde et al. 2020a). In recent outlines of Dothideomycetes (Hongsanan et al. 2020; Pem et al. 2020; Wijayawardene et al. 2020), 14 genera have been accepted in Hysteriaceae.

*Rhytidhysteron* was introduced by Spegazzini (1881) to accommodate two species: *Rhytidhysteronbrasiliense* (type species) and *R.viride* in Patellariaceae (Clements and Shear 1931; Kutorga and Hawksworth 1997). Boehm et al. (2009a, b) transferred *Rhytidhysteron* from Patellariaceae to Hysteriaceae based on molecular data. Subsequent studies introduced more taxa and records in *Rhytidhysteron* with both morphological and molecular evidence (Thambugala et al. 2016; Doilom et al. 2017; Cobos-Villagrán et al. 2020; Dayarathne et al. 2020; de Silva et al. 2020; Hyde et al. 2020a, b; Mapook et al. 2020; Wanasinghe et al. 2021). Currently, 24 species are recognized in *Rhytidhysteron* (Species Fungorum 2021; Wanasinghe et al. 2021).

*Rhytidhysteron* species have been documented from a wide range of hosts in various countries such as Australia, Bermuda, Bolivia, Brazil, China, Colombia, Cuba, France, Hawaii, India, New Zealand, Thailand, Ukraine, USA, and Venezuela (Kutorga and Hawksworth 1997; de Silva et al. 2020). Most *Rhytidhysteron* species are identified as saprobes on woody-based substrates in terrestrial habitats as well as from mangrove wood in marine habitats (Thambugala et al. 2016; Kumar et al. 2019; Hyde et al. 2020a, b; Wanasinghe et al. 2021). However, they have also been reported as endophytes or weak pathogens on woody plants and seldom as human pathogens (Soto and Lucking 2017; de Silva et al. 2020). From a biotechnological perspective, *Rhytidhysteron* species have great potential for their commercial applications and in industry. In particular, interest in secondary metabolites has rekindled in recent years, for instance with the discovery of palmarumycins. The latter is a potential inhibitor of thioredoxin–thioredoxin reductase cellular redox systems, with potential antimicrobial and antifungal properties (Murillo et al. 2009). Other *Rhytidhysteron* species discovered from the Southeast Asian region, such as *R.bruguierae* (MFLUCC 17-1515) and *R.chromolaenae* (MFLUCC 17-1516) also showed antimicrobial activity against *Mucorplumbeus* (Mapook et al. 2020) and hence this demonstrates a potential biotechnological application.

The Greater Mekong Subregion (GMS) is regarded as a global biodiversity hotspot due to its widely varying environmental conditions. Accordingly, the GMS harbors a diverse array of numerous florae, fauna and microorganisms (Li et al. 2018). Woody litter microfungi is an overlooked group of fungi in GMS and based on previous fungal estimates, there is undoubtedly a large number of new species yet to be described from this region. Our ongoing studies into the diversity of microfungi of the GMS are actively contributing towards filling in the knowledge gap in fungal taxonomy, phylogeny, host association and ecological distribution of *Rhytidhysteron* species in this region (Luo et al. 2018; Bao et al. 2019; Dong et al. 2020; Hyde et al. 2020b; Monkai et al. 2020, 2021; Wanasinghe et al. 2020, 2021; Yasanthika et al. 2020). Our specific objectives of this study are as follows: 1) to describe a novel species of *Rhytidhysteron* with evidence from morphology and DNA sequence data; 2) to characterize (based on morphology and phylogeny) additional new records of *Rhytidhysteron*; 3) to investigate the phylogenetic relationships of our *Rhytidhysteron* samples based on DNA sequence analyses from rDNA and protein coding genes and update the taxonomy of *Rhytidhysteron*.

## ﻿Materials and methods

### ﻿Samples collection and morphological analyses

Woody litter samples were collected from China (Kunming, Yunnan Province) during the wet season (August 2019) and during the dry season (December 2019) collections were done in Thailand (Chiang Rai and Tak Provinces). Samples were brought to the laboratory in plastic Ziploc bags. Fungal specimens were then examined using a stereomicroscope (Olympus SZ61, China). Pure cultures were obtained via single spore isolation on potato dextrose agar (PDA) following the methods described in Senanayake et al. (2020). Cultures were incubated at 25 °C for one week in the dark. Digital images of the fruiting structures were captured with a Canon (EOS 600D) digital camera fitted to a Nikon ECLIPSE Ni compound microscope. Squash mount preparations were prepared to determine micro-morphology and free hand sections of sporocarps made to observe the shapes of ascomata/conidiomata and peridium structures. Measurements of morphological structures were taken from the widest part of each structure. When possible, more than 30 measurements were made. Measurements were taken using the Tarosoft (R) Image Frame Work program. Figures were processed using Adobe Photoshop CS6. Field data are presented in ‘Material examined’. Other details pertaining to good practices of morphological examinations were done following guidelines by Senanayake et al. (2020). New species are established based on recommendations proposed by Jeewon and Hyde (2016). Type specimens were deposited in the herbarium of the Cryptogams Kunming Institute of Botany Academia Sinica (KUN-HKAS). Ex-type living cultures were deposited at the Culture Collection of Mae Fah Luang University (MFLUCC) and Kunming Institute of Botany Culture Collection (KUMCC).

### ﻿DNA extraction, amplification and sequencing

Genomic DNA was extracted from the mycelium grown on PDA at 25–30 °C for one week using a Biospin Fungus Genomic DNA Extraction Kit (BioFlux Hangzhou, P. R. China). Three partial rDNA genes and a protein coding gene were processed in our study, including the small ribosomal subunit RNA (SSU) using the primer pair NS1/NS4 (White et al. 1990), internal transcribed spacer region (ITS) using the primer pair ITS5/ITS4 (White et al. 1990), large nuclear ribosomal subunit (LSU) using primer pair LR0R/LR5 (Vilgalys and Hester 1990), translation elongation factor 1-alpha gene (*tef*1-α) using primer pair 983F/2218R (Rehner and Buckley 2005). Amplification reactions were performed in a total volume of 25 μL of PCR mixtures containing 8.5 μL ddH_2_O, 12.5 μL 2X PCR MasterMix (TIANGEN Co., China), 2 μL DNA template and 1 μL of each primer. PCR thermal cycle program for SSU, LSU, ITS, and *tef*1-α were set as described in Wanasinghe et al. (2020). The PCR products were sent to the Qingke Company, Kunming City, Yunnan Province, China, for sequencing. Sequences were deposited in GenBank (Table 1).

**Table 1.** GenBank accession numbers of sequences used for the phylogenetic analyses.

Taxon	Strain number	GenBank accession numbers				Reference
		SSU	LSU	ITS	*tef*1-α	
*Gloniopsiscalami*	MFLUCC 15-0739	KX669034	NG_059715	KX669036	KX671965	Hyde et al. (2016)
*Gloniopsispraelonga*	CBS 112415	FJ161134	FJ161173	NA	FJ161090	Boehm et al. (2009a)
*Rhytidhysteronbruguierae*	MFLUCC 18-0398^T^	MN017901	MN017833	NA	MN077056	Dayarathne et al. (2020)
*Rhytidhysteronbruguierae*	MFLUCC 17-1515	MN632463	MN632452	MN632457	MN635661	Mapook et al. (2020)
*Rhytidhysteronbruguierae*	MFLUCC 17 1511	MN632465	MN632454	MN632459	NA	Mapook et al. (2020)
*Rhytidhysteronbruguierae*	MFLUCC 17-1502	MN632464	MN632453	MN632458	MN635662	Mapook et al. (2020)
*Rhytidhysteronbruguierae*	MFLUCC 17-1509	MN632466	MN632455	MN632460	NA	Mapook et al. (2020)
*Rhytidhysteroncamporesii*	HKAS 104277^T^	NA	MN429072	MN429069	MN442087	Hyde et al. (2020a)
*Rhytidhysteronchromolaenae*	MFLUCC 17-1516^T^	MN632467	MN632456	MN632461	MN635663	Mapook et al. (2020)
*Rhytidhysteronerioi*	MFLU 16-0584^T^	NA	MN429071	MN429068	MN442086	Hyde et al. (2020a)
*Rhytidhysteronhongheense*	KUMCC 20-0222^T^	MW264224	MW264194	MW264215	MW256816	Wanasinghe et al. (2021)
*Rhytidhysteronhongheense*	HKAS112348	MW541831	MW541820	MW54182	MW556132	Wanasinghe et al. (2021)
*Rhytidhysteronhongheense*	HKAS112349	MW541832	MW541821	MW541825	MW556133	Wanasinghe et al. (2021)
*Rhytidhysteronhysterinum*	EB 0351	NA	GU397350	NA	GU397340	Boehm et al. (2009b)
*Rhytidhysteronmagnoliae*	MFLUCC 18-0719^T^	MN989382	MN989384	MN989383	MN997309	de Silva et al. (2020)
*Rhytidhysteronmangrovei*	MFLUCC 18-1113^T^	NA	MK357777	MK425188	MK450030	Kumar et al. (2019)
*Rhytidhysteronneorufulum*	MFLUCC 13-0216^T^	KU377571	KU377566	KU377561	KU510400	Thambugala et al. (2016)
*Rhytidhysteronneorufulum*	GKM 361A	GU296192	GQ221893	NA	NA	Thambugala et al. (2016)
*Rhytidhysteronneorufulum*	HUEFS 192194	NA	KF914915	NA	NA	Thambugala et al. (2016)
*Rhytidhysteronneorufulum*	MFLUCC 12-0528	KJ418119	KJ418117	KJ418118	NA	Thambugala et al. (2016)
*Rhytidhysteronneorufulum*	CBS 306.38	AF164375	FJ469672	NA	GU349031	Thambugala et al. (2016)
*Rhytidhysteronneorufulum*	MFLUCC 12-0011	KJ418110	KJ418109	KJ206287	NA	Thambugala et al. (2016)
*Rhytidhysteronneorufulum*	MFLUCC 12-0567	KJ546129	KJ526126	KJ546124	NA	Thambugala et al. (2016)
*Rhytidhysteronneorufulum*	MFLUCC 12-0569	KJ546131	KJ526128	KJ546126	NA	Thambugala et al. (2016)
*Rhytidhysteronneorufulum*	EB 0381	GU397366	GU397351	NA	NA	Thambugala et al. (2016)
***Rhytidhysteronneorufulum***	**MFLUCC 21-00**35	**MZ346025**	**MZ346015**	**MZ346020**	**MZ356249**	**This study**
*Rhytidhysteronopuntiae*	GKM 1190	NA	GQ221892	NA	GU397341	Mugambi et al. (2009)
*Rhytidhysteronrufulum*	MFLUCC 14-0577^T^	KU377570	KU377565	KU377560	KU510399	Thambugala et al. (2016)
*Rhytidhysteronrufulum*	EB 0384	GU397368	GU397354	NA	NA	Boehm et al. (2009b)
*Rhytidhysteronrufulum*	EB 0382	NA	GU397352	NA	NA	Boehm et al. (2009b)
*Rhytidhysteronrufulum*	EB 0383	GU397367	GU397353	NA	NA	Boehm et al. (2009b)
*Rhytidhysteronrufulum*	MFLUCC 12-0013	KJ418113	KJ418111	KJ418112	NA	de Silva et al. (2020)
*Rhytidhysterontectonae*	MFLUCC 13-0710^T^	KU712457	KU764698	KU144936	KU872760	Doilom et al. (2017)
***Rhytidhysterontectonae***	**MFLUCC 21-0037**	**MZ346023**	**MZ346013**	**MZ346018**	**MZ356247**	**This study**
***Rhytidhysterontectonae***	**MFLUCC 21-0034**	**MZ346024**	**MZ346014**	**MZ346019**	**MZ356248**	**This study**
*Rhytidhysteronthailandicum*	MFLUCC 14-0503^T^	KU377569	KU377564	KU377559	KU497490	Thambugala et al. (2016)
*Rhytidhysteronthailandicum*	MFLUCC 12-0530	KJ546128	KJ526125	KJ546123	NA	Thambugala et al. (2016)
*Rhytidhysteronthailandicum*	MFLU17-0788	MT093495	MT093472	MT093733	NA	de Silva et al. (2020)
***Rhytidhysteronxiaokongense***	**KUMCC 20-0158**	**MZ346021**	**MZ346011**	**MZ346016**	**MZ356245**	**This study**
***Rhytidhysteronxiaokongense***	**KUMCC 20-0160^T^**	**MZ346022**	**MZ346012**	**MZ346017**	**MZ356246**	**This study**

Ex-type strains are indicated with superscript “T”, and newly generated sequences are shown in bold. NA represents sequences that are unavailable in GenBank.

### ﻿Phylogenetic analyses

Representative species used in the phylogenetic analyses were selected based on previous publications (Thambugala et al. 2016; Mapook et al. 2020; Wanasinghe et al. 2021). Sequences were downloaded from GenBank (http://www.ncbi.nlm.nih.gov/) and their accession numbers are listed in Table 1. The newly generated sequences in this study were assembled by BioEdit 7.0.9.0 (Hall 1999). Individual gene regions were separately aligned in MAFFT v.7 web server (http://mafft.cbrc.jp/alignment/server/) (Katoh et al. 2019). The alignments of each gene were improved by manually deleting the ambiguous regions and gaps, and then combined using BioEdit 7.2.3. Final alignments containing SSU, LSU, ITS, an*d tef*1-α were converted to NEXUS format (.nxs) using CLUSTAL X (2.0) and PAUP v. 4.0b10 (Thompson et al. 1997; Swofford 2002) and processed for Bayesian and maximum parsimony analysis. The FASTA format was changed into PHYLIP format via the Alignment Transformation Environment (ALTER) online program (http://www.sing-group.org/ALTER/) and used for maximum likelihood analysis (ML).

**Figure 1.**
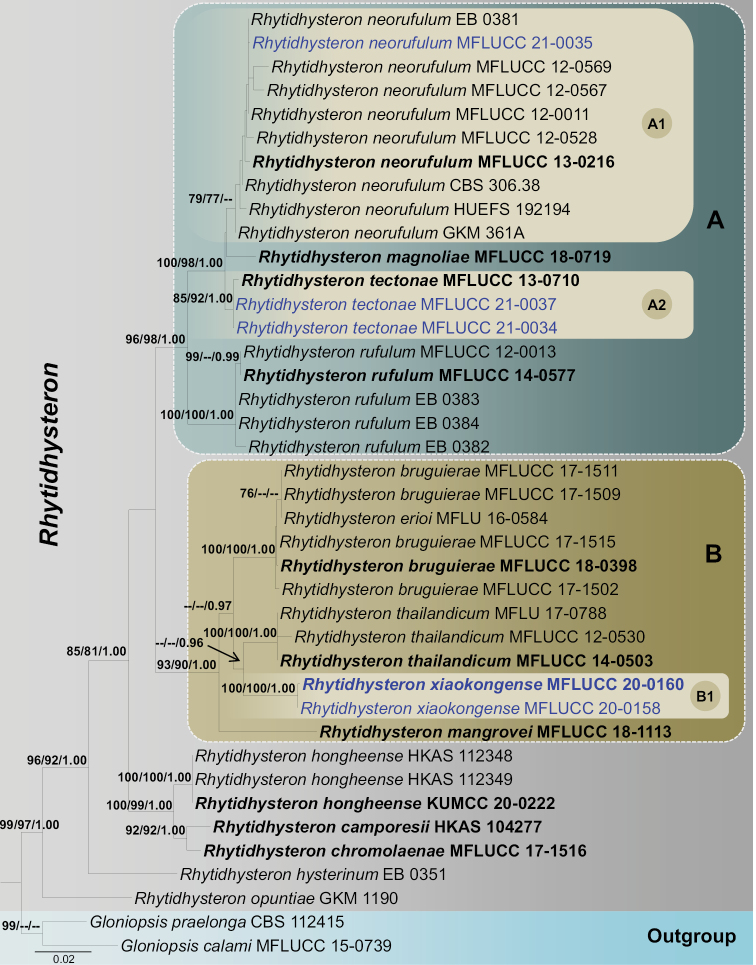
RAxML tree based on a combined dataset of partial SSU, LSU, ITS, and *tef*1-α sequence analyses. Bootstrap support values for ML and MP equal to or higher than 75% and Bayesian PP equal to or greater than 0.95 are shown at the nodes. Hyphens (--) represent support values less than 75% / 0.95 BYPP. The ex-type strains are in bold and the new isolate in this study is in blue. The tree is rooted with *Gloniopsiscalami* (MFLUCC 15-0739) and *G.praelonga* (CBS 112415).

ML was carried out in CIPRES Science Gateway v.3.3 (http://www.phylo.org/portal2/; Miller et al. 2010) using RAxML-HPC2 on XSEDE (8.2.12) (Stamatakis 2014) with the GTRGAMMA substitution model and 1,000 bootstrap iterations. Maximum parsimony analysis (MP) was performed in PAUP v. 4.0b10 (Swofford 2002) with the heuristic search option and Tree-Bisection-Reconnection (TBR) of branch-swapping algorithm for 1,000 random replicates. Branches with a minimum branch length of zero were collapsed and gaps were treated as missing data (Hillis and Bull 1993). ML and MP bootstrap values (ML) ≥ 75% are given above each node of the phylogenetic tree (Fig. 1).

Bayesian analysis was executed in MrBayes v.3.2.2 (Ronquist et al. 2012). The model of evolution was estimated using MrModeltest v. 2.3 (Nylander et al. 2008) via PAUP v. 4.0b10 (Ronquist and Huelsenbeck 2003). The HKY+I for SSU; GTR+I+G for ITS, LSU and *tef*1-α were used in the final command. Markov chain Monte Carlo sampling (MCMC) in MrBayes v.3.2.2 (Ronquist et al. 2012) was used to determine posterior probabilities (PP) (Rannala and Yang 1996; Zhaxybayeva and Gogarten 2002). Bayesian analyses of six simultaneous Markov chains were run for 2,000,000 generations and trees were sampled every 200 generations (resulting in 10,001 total trees). The first 25% of sampled trees were discarded as part of the burn-in procedure, the remaining 7,501 trees were used to create the consensus tree, and the average standard deviation of split frequencies was set as 0.01. Branches with Bayesian posterior probabilities (BYPP) ≥ 0.95 are indicated above each node of the phylogenetic tree (Fig. 1). Phylogenetic trees were visualized in FigTree v1.4.0 (http://tree.bio.ed.ac.uk/software/figtree/; Rambaut 2012). The tree was edited using Microsoft PowerPoint before being, then saved in PDF format and finally converted to JPG format using Adobe Illustrator CS6 (Adobe Systems, USA). The finalized alignments and trees were deposited in TreeBASE, submission ID: TB2:S28620 (http://purl.org/phylo/treebase/phylows/study/TB2:S28620).

## ﻿Results

### ﻿Phylogenetic analysis

The phylogenetic analysis was conducted using 38 strains in *Rhytidhysteron*, and two outgroup taxa viz. *Gloniopsiscalami* (MFLUCC 15-0739) and *G.praelonga* (CBS 112415) in Pleosporales (Table 1). The aligned sequence matrix comprised four gene regions (SSU: 1018 bp, LSU: 891 bp, ITS: 742 bp and *tef*1-α: 953 bp) and a total of 3,604 characters (including gaps), of which 3,095 characters were constant, 161 variable characters were parsimony-uninformative and 348 characters were parsimony-informative. The Kishino-Hasegawa test shows length = 928 steps with CI = 0.696, RI = 0.846, RC = 0.589 and HI = 0.304. The RAxML analysis of the combined dataset yielded a best-scoring tree with a final ML optimization likelihood value of -10181.226009. The matrix had 723 distinct alignment patterns, with 26.6% of undetermined characters or gaps. Estimated base frequencies were as follows: A = 0.242390, C = 0.244261, G = 0.276352, T = 0.236997; substitution rates AC = 1.457846, AG = 2.708684, AT = 1.298658, CG = 0.909442, CT = 6.323746, GT = 1.00; gamma distribution shape parameter α = 0.02.

Topologies of the phylogenetic trees under ML, MP and BI criteria recovered for each gene dataset were visually compared, and the overall tree topology was similar to those obtained from the combined dataset (Figure 1). Our analyzed molecular data generated phylogeny of *Rhytidhysteron* species was consistent with those of Wanasinghe et al. (2021). The maximum likelihood tree generated based on sequence analysis of the combined (ribosomal DNA: SSU, LSU and ITS; and protein coding gene: *tef*1-α) dataset recovered three major monophyletic clades within *Rhytidhysteron* (A-C, Figure 1) and two basal lineages *viz. R.hysterinum* (EB 0351) and *R.opuntiae* (GKM 1190). Clade A comprises *Rhytidhysteronmagnoliae*, *R.neorufulum*, *R.rufulum* and *R.tectonae* with 96% ML, 98% MP and 1.00 BYPP support values.

One of our new isolates, MFLUCC 21-0035 grouped with another nine *Rhytidhysteronneorufulum* strains (CBS 306.38, EB 0381, GKM 361A, HUEFS 192194, MFLUCC 12-0011, MFLUCC 12-0528, MFLUCC 12-0567, MFLUCC 12-0569, MFLUCC 13-0216, MFLUCC 21-0035). However, this relationship is not statistically supported in Bayesian analysis, retrieving 79% and 77% support values in ML and MP, respectively (sub clade A1, Figure 1). *Rhytidhysteronmagnoliae* (MFLUCC 18-0719) constitutes an independent lineage and is a sister taxon to others in sub clade A1, and this was not statistically supported.

Two newly generated sequences MFLUCC 21-0034 and MFLUCC 21-0037 grouped with the type strain of *Rhytidhysterontectonae* (MFLUCC 13-0710) as a monophyletic clade within Clade A (subclade A2, Figure 1). This association was supported by 85% ML, 92% MP and 1.00 BYPP bootstrap values (subclade A2, Figure 1). Five strains of *Rhytidhysteronrufulum* (EB 0382, EB 0383, EB 0384, MFLUCC 12-0013, MFLUCC 14-0577) constitute another strongly monophyletic group basal to Clade A.

Two of our newly generated sequences, *Rhytidhysteronxiaokongense* (KUMCC 20-0158, KUMCC 20-0160), grouped with *R.bruguierae* (MFLUCC 17-1511, MFLUCC 17-1502, MFLUCC 17-1509, MFLUCC 17-1515, MFLUCC 18-0398), *R.erioi* (MFLU 16-0584), *R.mangrovei* (MFLUCC 18-1113) and *R.thailandicum* (MFLU 17-0788, MFLUCC 12-0530, MFLUCC 14-0503). These taxa form a monophyletic clade (Clade B) in *Rhytidhysteron* with 93% ML, 91% MP and 1.00 BYPP bootstrap values. Within this clade (Clade B), *Rhytidhysteronxiaokongense* (KUMCC 20-0158 and KUMCC 20-0160) clusters together (subclade B1) with high bootstrap values (100% ML, 100% MP and 1.00 BYPP) and is sister to *Rhytidhysteronthailandicum*. However, the latter relationship was only supported by BI analysis with 0.96 BYPP.

*Rhytidhysteroncamporesii* (HKAS104277), *R.chromolaenae* (MFLUCC 17-1516) and *R.hongheense* (HKAS112348, HKAS112349, KUMCC 20-0222) grouped as a monophyletic clade. This relationship is statistically supported with 100% ML, 99% MP and 1.00 BYPP values (Figure 1). *Rhytidhysteronhysterinum* (EB 0351) and *R.opuntiae* (GKM 1190) nested as basal lineages in *Rhytidhysteron* (Figure 1).

## ﻿Taxonomy

### ﻿ Rhytidhysteron xiaokongense

Taxon classification
Fungi
Patellariales
Patellariaceae


G.C. Ren & K.D. Hyde sp. nov.

87692543-D03E-5EF3-BDED-7D2E30016BF7

558453

Facesoffungi Number No: FoF09903

Figure 2

#### Etymology.

The species epithet reflects the location where the species was collected.

#### Holotype.

HKAS 112728.

#### Diagnosis.

Similar to *R.hysterinum* and *R.rufulum*, but differs in some conidial features.

#### Description.

*Saprobic* on woody litter of *Prunus* sp. **Sexual morph** Undetermined. **Asexual morph***Conidiomata* 448–464 × 324–422 µm (x̄ = 454 × 378 μm, n = 5), solitary, scattered, semi-immersed in the host, black, unilocular, subglobose to ampulliform. *Ostioles* 178–227 × 166–234 µm (x̄ = 205 × 198 μm, n = 6), central, short papillate. *Conidiomata wall* 30–40 μm thick, 4–6 layers, reddish-brown to dark brown cells of *textura angularis*. *Conidiogenous cells* 5–8 × 3–6 µm (x̄ = 6.8 × 4.5 μm, n = 10), subglobose or ellipsoidal, hyaline, smooth, forming in a single layer over the entire inner surface of the wall, discrete, producing a single conidium at the apex. *Conidia* 20–25 × 8–10 μm (x̄ = 22 × 9 μm, n = 20), hyaline to yellowish-brown when immature, becoming brown to dark brown at maturity, oblong to ellipsoidal, with rounded ends, straight to slightly curved, aseptate when immature, becoming 1-septate when mature, with granular appearance, slightly constricted at septa.

#### Habitat and distribution.

Known to inhabit woody litter of *Prunus* sp. (Yunnan, China) (this study).

#### Material examined.

China, Yunnan Province, Kunming city, Xiaokong Mountain (25.171311°N, 102.703690°E), on dead wood of *Prunus* sp. (Rosaceae), 21-Dec-2019, G.C. Ren, KM18 (HKAS 112728, holotype), ex-type living culture KUMCC 20-0160; KM17 (HKAS 112727, paratype), ex-paratype living culture KUMCC 20-0158.

#### Notes.

*Rhytidhysteronxiaokongense* is similar to *R.hysterinum* and *R.rufulum* in having black, unilocular, subglobose conidiomata and dark brown, 1-septate conidia. However, some of the conidia features in these species are different: *R.xiaokongense* has oblong to ellipsoidal conidia with rounded ends, whereas the conidia of *R.rufulum* and *R.hysterinum* have a truncated base with a pore in the middle of the septum (Samuels and Müller 1979). In the phylogenetic analyses, *R.xiaokongense* is distinct from *R.rufulum* and *R.hysterinum* and is more closely related to *R.thailandicum*. *Rhytidhysteronxiaokongense* has 1-septate, dark brown, oblong to ellipsoidal conidia, while *R.thailandicum* has globose to subglobose, hyaline conidia (Thambugala et al. 2016). The sequence data from both mycelium and fruiting bodies confirms that single spore isolation was successfully performed.

**Figure 2.**
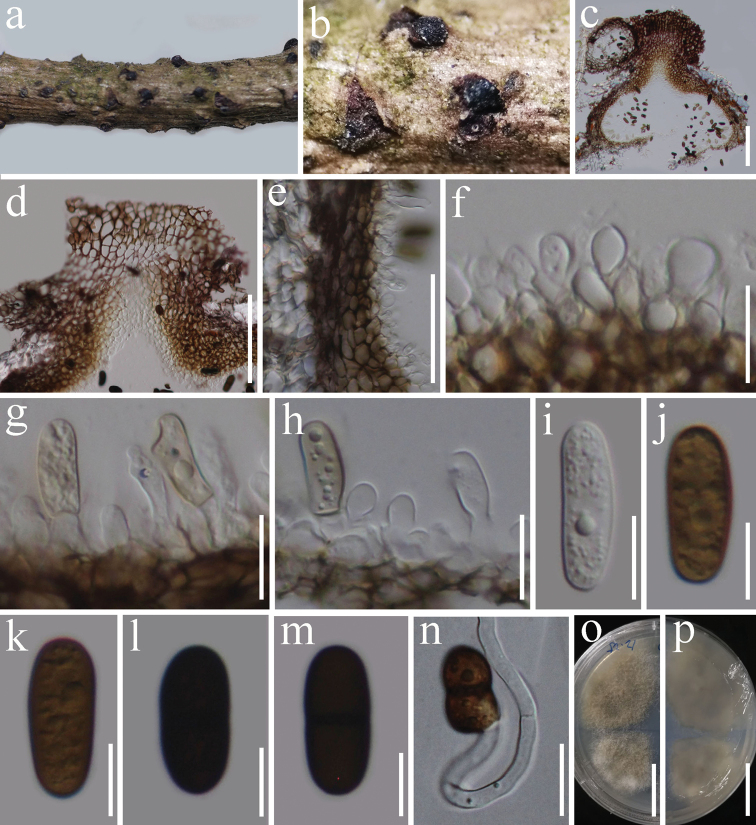
*Rhytidhysteronxiaokongense* (HKAS 112728, holotype) **a, b** conidiomata on natural wood surface **c** sections through conidioma **d** ostiolar neck **e** conidioma wall **f–h** conidiogenous cells and developing conidia **i–m** conidia **n** germinated conidium **o, p** culture characters on PDA (**o** = above, **p** = reverse). Scale bars: 100 μm (**c, d**); 50 μm (**e**); 15 μm (**f–h**); 10 μm (**i–m**); 20 μm (**n**); 25 mm (**o, p**).

### ﻿ Rhytidhysteron tectonae

Taxon classification
Fungi
Patellariales
Patellariaceae


Doilom & K.D. Hyde, Fungal Diversity. 82: 107–182 (2017)

A040E115-BF8E-5F4A-B91B-BCF1737464B4

551964

Facesoffungi number No: FoF01849

Figure 3

#### Description.

*Saprobic* on decaying wood. **Sexual morph***Hysterothecia* 550–950 µm long, 450–600 µm high, 400–500 diam. (x̄ = 800 × 500 × 450 µm, n = 5), semi-immersed to superficial, scattered, apothecial, erumpent from the substrate, dark brown to black, coriaceous, elongate with a longitudinal slit. *Exciple* 70–110 µm (x̄ = 90 µm, n = 15), thick-walled, composed of brown to dark brown cells of *textura globulosa* to *angularis*. *Hamathecium* comprising 1–2 μm wide, numerous, septate, branched, pseudoparaphyses. *Asci* 170–200 × 10–12 μm (x̄ = 190 × 11, n = 15), 8-spored, bitunicate, cylindrical, with short pedicel, rounded at the apex, with an ocular chamber. *Ascospores* 25–29 × 8–10 µm (x̄ = 27 × 9 µm, n = 20), uniseriate, hyaline to brown, 1–3-septate, smooth-walled, ellipsoidal to fusoid, straight or curved, rounded to slightly pointed at both ends, guttulate. **Asexual morph** Undetermined.

**Figure 3.**
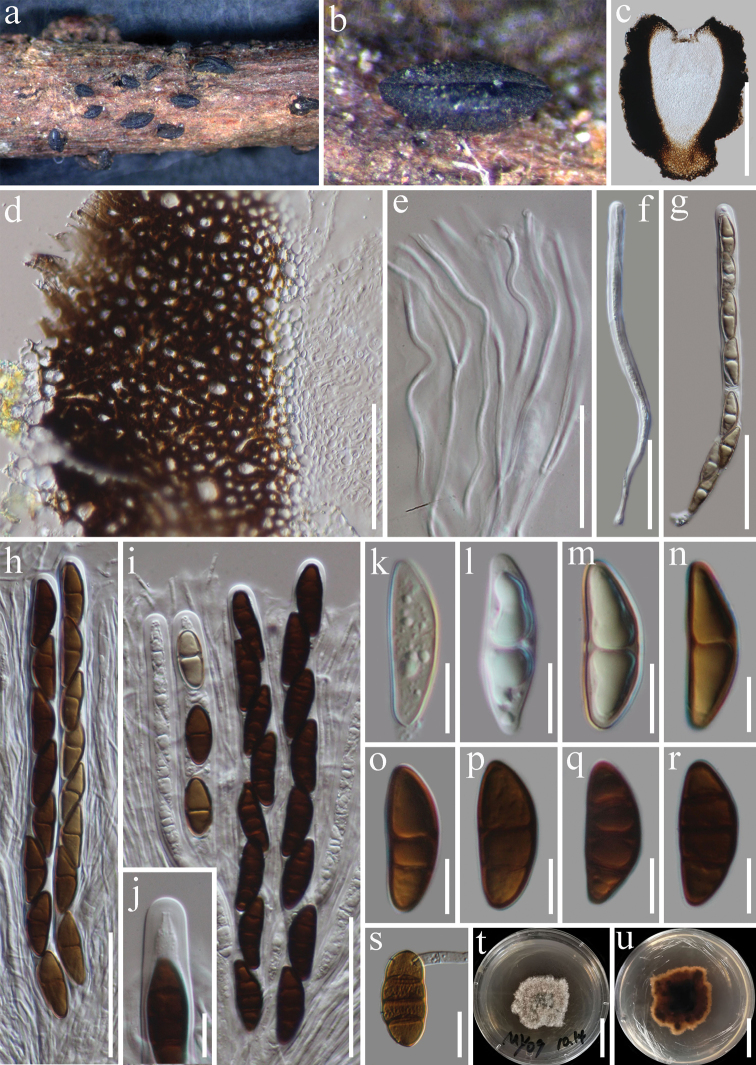
*Rhytidhysterontectonae* (HKAS 115533) **a, b***Hysterothecium* on wood **c** vertical section through hysterothecia **d** exciple **e** pseudoparaphyses **f–i** immature and mature asci **j** ocular chamber. **k–r** immature and mature ascospores **s** Germinating ascospore **t, u** culture characters on PDA (**t** = above view, **u** = reverse view). Scale bars: 300 μm (**c**); 50 μm (**d**); 30 μm (**e**); 50 μm (**f–i**); 10 μm (**j–r**); 15 μm (**s**); 25 mm (**t, u**).

#### Habitat and distribution.

Known to inhabit dead branches of *Tectonagrandis*, *Betula* sp. (Betulaceae) and Fabaceae sp (Thailand) (Doilom et al. 2017; this study).

#### Material examined.

Thailand, Chiang Rai Province, Mae Yao District, on dead woody twigs of *Betula* sp. (Betulaceae), 23-Sep-2019, G.C. Ren, MY09 (HKAS 115533), living culture MFLUCC 21-0037; Thailand, Chiang Rai Province, Mae Fah Luang University, on dead woody twigs of Fabaceae, 5-Jul-2019, G.C. Ren, RMFLU19001 (HKAS 115532), living culture MFLUCC 21-0034.

#### Notes.

*Rhytidhysterontectonae* was introduced by Doilom et al. (2017) based on morphological and phylogenetic analyses from dead branches of *Tectonagrandis* in Thailand. Based on our phylogenetic analysis of the combined SSU, LSU, ITS, and *tef*1-α sequence data, our collections (MFLUCC 21-0034 and MFLUCC 21-0037) cluster with the strain of *R.tectonae* (MFLUCC 13-0710) with 85% ML, 92% MP, 1.00 PP bootstrap support (Figure 1). Our collection shares similar morphological features with *R.tectonae* (MFLU 14-0607). However, our new collection has smaller hysterothecia (800 × 500 × 450 μm vs 2175 × 585 × 523 μm) and longer asci (190 μm vs 155 μm) in comparison to the type. Based on morphological characteristics and phylogenetic analysis, we introduce MFLUCC 21-0034 and MFLUCC 21-0037 as new host records of *R.tectonae* from decaying wood of *Betula* sp. and Fabaceae sp. in Thailand.

### ﻿ Rhytidhysteron neorufulum

Taxon classification
Fungi
Patellariales
Patellariaceae


Thambug. & K.D. Hyde, Cryptog. Mycol. 37(1): 110 (2016)

52DFAE6A-942F-58F9-944E-34EB9C0FC19E

551865

Facesoffungi number No: FoF01840

Figure 4

#### Description.

*Saprobic* on decaying wood of *Tectonagrandis*. **Sexual morph***Hysterothecia* 1400–2100 μm long, 350–500 μm high, 600–1000 μm diam. (x̄ = 1780 × 400 × 700 μm, n = 5), superficial, black, solitary to aggregated, coriaceous, smooth, elliptical or irregular in shape, elongated with a longitudinal slit. *Exciple* 75–115μm (x̄ = 90, n = 20) wide, composed of several layers of brown to dark brown, thick-walled cells of *textura angularis*. *Hamathecium* 2–3.5 μm wide, dense, septate pseudoparaphyses, constricted at the septum, filiform, pale-yellow pigmented, forming epithecium above the asci and enclosed in a gelatinous matrix. *Asci* 190–260 × 13–18 μm (x̄ = 230 × 16 μm, n = 10), 8-spored, bitunicate, clavate to cylindrical, with a short furcate pedicel, apically rounded, without a distinct ocular chamber. *Ascospores* 36–44 × 11–17 μm (x̄ = 41 × 13 μm, n = 30), uni-seriate, yellowish to brown, with 1–3-septa, ellipsoidal to fusiform, slightly rounded or pointed at both ends, constricted at the central septum, with granular appearance. **Asexual morph** Undetermined.

**Figure 4.**
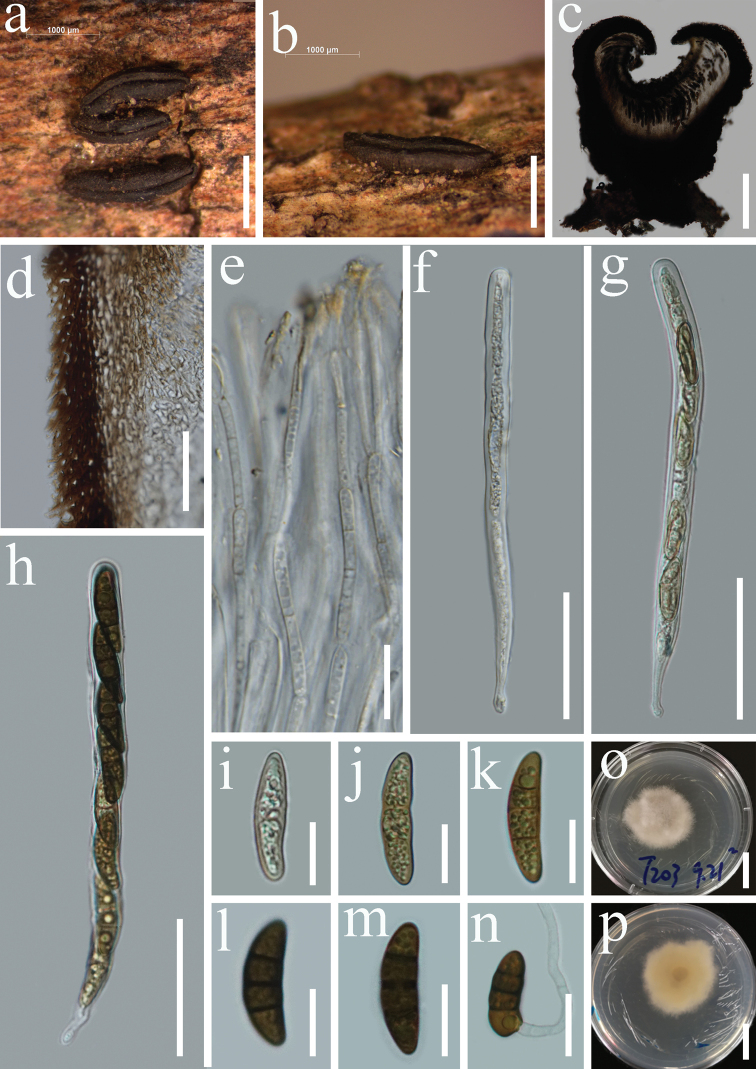
*Rhytidhysteronneorufulum* (HKAS 115534) **a, b***Hysterothecium* on wood **c** vertical section through hysterothecia **d** exciple **e** pseudoparaphyses **f–h** immature asci and mature asci **i–m** immature ascospores and mature ascospores **n** germinating ascospore **o, p** culture characters on PDA (**o** = above view, **p** = reverse view). Scale bars: 1000 μm (**a, b**); 200 μm (**c**); 15 μm (**d**); 20 μm (**e**); 50 μm (**f–h**); 10 μm (**i–m**); 20 μm (**n**); 20 mm (**o, p**).

#### Habitat and distribution.

*Bursera* sp (Mexico), *Heveabrasiliensis* and *Tectonagrandis* (Thailand) (Thambugala et al. 2016; Cobos-Villagran et al. 2020; this study).

#### Material examined.

Thailand, Tak Province, Mogro District, Amphoe Umphang, on dead woods of *Tectonagrandis* (Lamiaceae), 20-Aug-2019, G.C. Ren, T203 (HKAS 115534), living culture MFLUCC 21-0035.

#### Notes.

*Rhytidhysteronneorufulum* was introduced by Thambugala et al. (2016) based on both morphological and phylogenetic analyses of a combined dataset of LSU, SSU and *tef*1-α sequence data. Thambugala et al. (2016) accounted *R.neorufulum* (MFLUCC 13-0216) from decaying woody stems and twigs in Thailand. Our new collection shares similar morphology to that of the type description of *Rhytidhysteronneorufulum* (MFLUCC 13-0216) in having superficial, coriaceous, elliptical or irregular, elongated hysterothecia with a longitudinal slit, bitunicate, cylindrical, short furcate pedicel asci and yellowish to brown, ellipsoidal to fusiform ascospores with 1–3-septa (Thambugala et al. 2016). However, our new collection has larger asci (190–260 × 13–18 μm vs 185–220 × 9.5–13 μm) and ascospores (36–44 × 11–17 μm vs 19–31 × 8–13 μm) in comparison to the type of *Rhytidhysteronneorufulum* (MFLUCC 13-0216). The multi-gene phylogenetic analysis based on combined SSU, LSU, ITS, and *tef*1-α sequence data showed that our collection is related to *Rhytidhysteronneorufulum* (Figure 1).

### ﻿Key to asexual morphs of *Rhytidhysteron* species

**Table:** 

1	Asexual morph has two types of conidia	**2**
–	Asexual morph has only one type of conidia	**3**
2	Comprising paraphyses	***R.hysterinum***
–	Paraphyses are absent	***R.rufulum***
3	Diplodia-like conidia	***R.xiaokongense***
–	Aposphaeria-like conidia	***R.thailandicum***

## ﻿Discussion

*Rhytidhysteron* is one of the first genera that trainee mycologists working on microfungi find in nature, as the hysterothecia are conspicuous (Hyde et al. 2020a). Species also easily germinate in culture and can easily be sequenced (Hyde et al. 2020a). Thus, it is even more remarkable that we found a new species in this study, indicating we are far from finding all species in this genus, and that more collections need be done on other continents (Hyde et al. 2020c). Most of *Rhytidhysteron* species are saprobes, which are essential for ecosystems functioning in terrestrial habitats and are commonly recognized as key biotic agents of wood decomposition, playing a vital role in carbon and nitrogen cycling in arid ecosystems, soil stability, plant biomass decomposition, and endophytic interactions with plants (Lustenhouwer et al. 2020; Dossa et al. 2021). Furthermore, *Rhytidhysteron* species have numerous antimicrobial and antifungal applications (Murillo et al. 2009; Mapook et al. 2020), and the discovery of new species provides new resources for future applied research in the field of biotechnology and industry.

Since the genus was established in 1881, a total of 24 species have been found to date, and the most commonly encountered species are *Rhytidhysteronneorufulum* and *R.rufulum*, so it might be difficult for mycologists to find new species within *Rhytidhysteron. Rhytidhysteron* is mainly identified via its sexual morph (Dayarathne et al. 2020; de Silva et al. 2020; Hyde et al. 2020a, b; Mapook et al. 2020; Wanasinghe et al. 2021). The asexual morphs of *Rhytidhysteron* have been reported as aposphaeria-like or diplodia-like, including *R.hysterinum* and *R.rufulum* (Samuels and Müller 1979). Thambugala et al. (2016) confirmed the asexual-sexual morph connection for *R.thailandicum* by aposphaeria-like asexual morphs forming in culture on PDA. Herein, we found a diplodia-like asexual morph of *Rhytidhysteron* from woody litter of *Prunus* sp. in China. In comparison to the occurrence of the sexual morph of *Rhytidhysteron*, asexual morphs seldom form under natural conditions. The discovery of this new species provides an important reference for the study of the asexual morphs of *Rhytidhysteron*. Moreover, findings from this study further enrich GMS*Rhytidhysteron* species diversity.

In our phylogenetic analyses, the new species, *Rhytidhysteronxiaokongense* was basal to *R.thailandicum* (Fig. 1). Although species in *Rhytidhysteron* are morphologically similar, our new species is an asexual form of the species found in nature, so it is easy to distinguish from other speices excluding the asexual forms of *R.hysterinum*, *R.rufulum* and *R.thailandicum*. *Rhytidhysteronxiaokongense* shares similar morphological characters to *R.hysterinum* and *R.rufulum* in having black, unilocular, subglobose conidiomata and dark brown, 1-septate conidia but conidial features differ (Samuels and Müller 1979). *Rhytidhysteronthailandicum* can be differentiated from *R.xiaokongense* with respects to its globose to subglobose, hyaline conidia (Thambugala et al. 2016). To further support the establishment of the new taxon as proposed by Jeewon and Hyde (2016), we examined the nucleotide differences within the ITS regions (ITS1-5.8S-ITS2) gene region. Comparison of the 507 nucleotides across the ITS regions reveals 39 bp (7.7%) differences between *Rhytidhysteronthailandicum* and *R.xiaokongense*.

*Rhytidhysteron* species are widely distributed throughout the globe (de Silva et al. 2020); however, they appear to be particularly abundant in Asia, where they are well studied. There is an abundance of species and collections in the Greater Mekong Subregion (China and Thailand), such as *R.brasiliense*, *R.camporesii*, *R.chromolaenae*, *R.erioi*, *R.hongheense*, *R.hysterinum*, *R.magnoliae*, *R.mangrovei*, *R.neorufulum*, *R.tectonae* and *R.thailandicum* (Thambugala et al. 2016; Doilom et al. 2017; Soto-Medina et al. 2017; Kumar et al. 2019; Cobos-Villagran et al. 2020; Dayarathne et al. 2020; de Silva et al. 2020; Hyde et al. 2020a; Mapook et al. 2020; Wanasinghe et al. 2021). We provide morphological and phylogenetic data for three species of *Rhytidhysteron* collected from the Greater Mekong Subregion: one new species, *Rhytidhysteronxiaokongense*, as a geographical record from China, two new host records of *R.tectonae* from woody litter of *Betula* sp and Fabaceae sp, and one new host record of *R.neorufulum* from woody litter of *Tectonagrandis*. Based on our current work and that of past studies (de Silva et al. 2020; Hyde et al. 2020a, b; Mapook et al. 2020; Wanasinghe et al. 2021), it is clear that species within *Rhytidhysteron* are likely cosmopolitan and not host-specific, with evidence of the same species being found on a number of different hosts. Importantly, the morphology of a single species sometimes shows slight variations under different environmental conditions, geographical regions, hosts and different life modes (Senanayake et al. 2020). It is therefore crucial to collect more species of *Rhytidhysteron* across different geographic regions and hosts, obtain more cultures and sequence data, and describe their morphology to improve knowledge of taxonomy and phylogeny.

## Supplementary Material

XML Treatment for Rhytidhysteron xiaokongense

XML Treatment for Rhytidhysteron tectonae

XML Treatment for Rhytidhysteron neorufulum
